# Spontaneous isolated celiac artery dissection: a rare mimicker of pancreatic mass masquerading as neoplasm

**DOI:** 10.1055/a-2780-9670

**Published:** 2026-02-13

**Authors:** Muhammad Umair Khalid, Hassan Siddiki

**Affiliations:** 1 583189 Gastroenterology, Hepatology, and Nutrition, Cleveland Clinic Foundation, Cleveland, Ohio, United States

A 77-year-old woman presented with a 3-month history of intermittent, nonradiating epigastric pain that became persistent and more severe over the 2 days prior to admission.

**Fig. 1:**
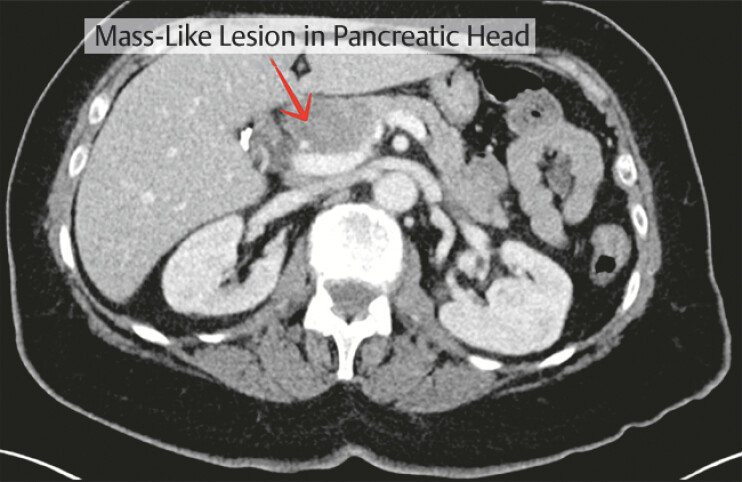
Initial abdominal CT demonstrating a mass-like lesion in the pancreatic head. CT, computed tomography.

**Fig. 2:**
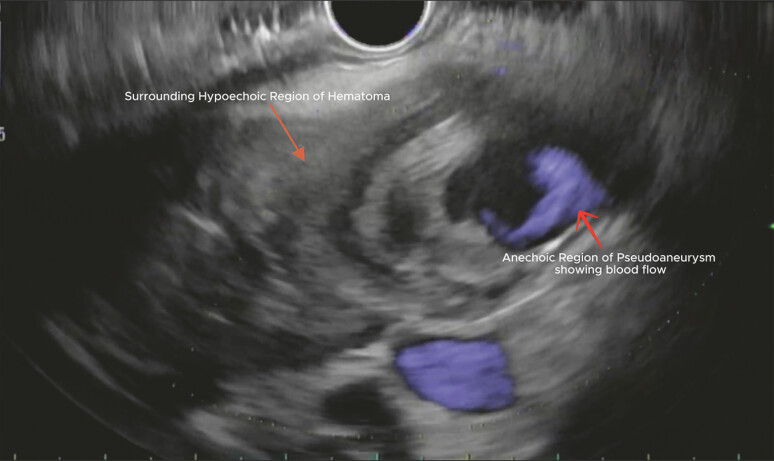
Endoscopic ultrasound (EUS) with Doppler showing hypoechoic hematoma with a central anechoic pseudoaneurysm with blood flow.

Initial computed tomographic (CT) imaging demonstrated a mass-like lesion in the pancreatic head which raised the concern for a pancreatic neoplasm ( Fig. 1 ). Advanced endoscopy was subsequently performed for further evaluation with endoscopic ultrasound (EUS) ( Video 1 ). EUS revealed an intimal tear in the celiac artery. A heterogeneous hypoechoic lesion adjacent to the pancreatic head with an internal anechoic component ( Fig. 2 ) was also visualized. This characteristic “donut” appearance 1 was more consistent with a pseudoaneurysm surrounded by hematoma rather than a solid pancreatic mass.

Diagnosis and management of spontaneous isolated celiac artery dissection (SICAD) with endoscopic ultrasound features mimicking a pancreatic mass.Video 1

**Fig. 3:**
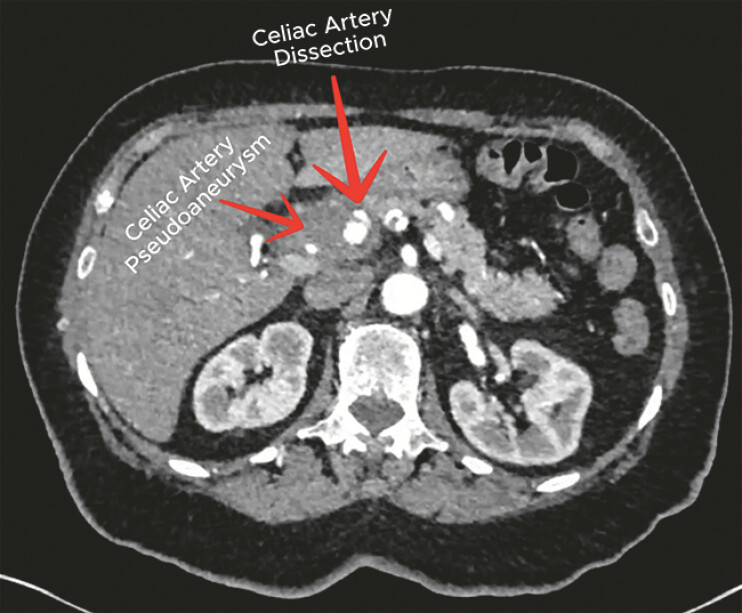
CT angiography depicting celiac artery dissection with an associated hematoma. CT, computed tomography.

**Fig. 4:**
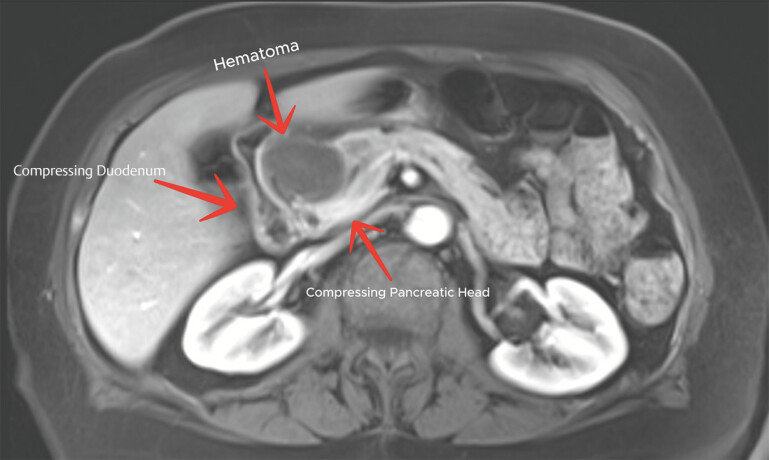
MRI demonstrating hematoma compressing duodenum and pancreatic head. MRI, magnetic resonance imaging.

Fine-needle aspiration was deferred due to the high risk of hemorrhage, and further vascular imaging was recommended. CT angiography confirmed a celiac artery dissection with an associated pseudoaneurysm at the celiac artery bifurcation ( Fig. 3 ). Magnetic resonance imaging further excluded a pancreatic mass and confirmed the aneurysm and dissection ( Fig. 4 ).

**Fig. 5:**
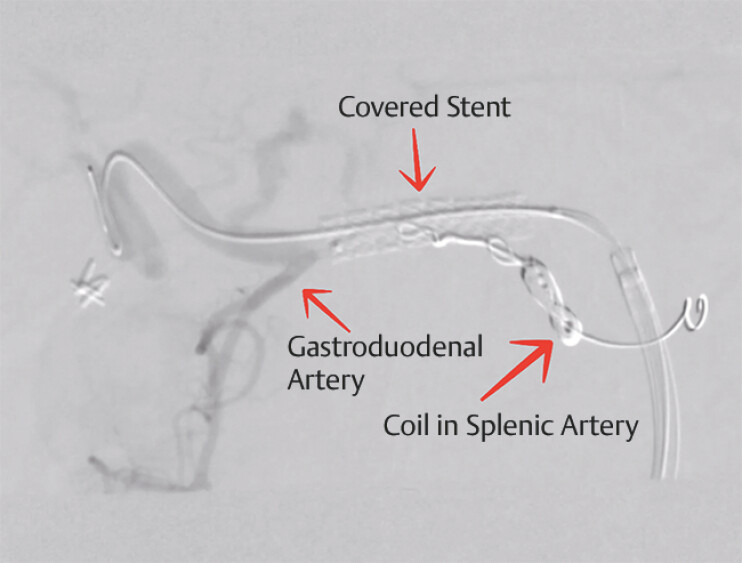
A final fluoroscopic image after endovascular management.

Vascular surgery subsequently managed the patient endovascularly. The splenic artery was embolized by a coil and a covered stent, extending from the abdominal aorta across the celiac trunk into the common hepatic artery, was placed ( Fig. 5 ). The patient had an uncomplicated post-operative course and was discharged the following day.

Spontaneous isolated celiac artery dissection (SICAD) is a rare condition which involves tear of the intima of the celiac artery without the involvement of aorta 2 . This case demonstrates key findings that the endoscopist should recognize to avoid missing a life-threatening diagnosis which needs urgent treatment and without treatment the patient can have catastrophic outcomes. CT angiography remains the most sensitive test for diagnoses 3 and standard CT with IV contrast can mischaracterize vascular lesions as masses, as in our case 4 .

Endoscopy_UCTN_Code_CCL_1AF_2AZ
